# Unusual relapse of primary central nervous system lymphoma

**DOI:** 10.1186/s40064-016-1926-x

**Published:** 2016-03-09

**Authors:** Marielle Igala

**Affiliations:** Hematology Department, Hôpital du 20 Aôut, Centre hospitalier universitaire de Casablanca, Casablanca, Morocco

**Keywords:** Primary central nervous system, Lymphoma, Liver, Relapse

## Abstract

Primary central nervous system lymphoma (PCNSL) is a rare disease which accounts for 1–2 % of non-Hodgkin lymphoma and 3–5 % of primary brain tumor lesions. PCNSL of an immunocompetent patient is an uncommon disease, it is estimated at 4 % of new diagnoses of CNS tumors. The prognosis of PCNSL is poor compared to other extranodal lymphomas, with a 5-year survival estimated between 20 and 40 %. PCNSL relapse occurs either in the original site but still confined to the CNS or exceptionally outside it. Brain magnetic resonance imaging, although not allowing a clear distinction between primary lesions and secondary brain lymphoma is of paramount importance not only for diagnosis but also for monitoring the patient. This manuscript report the case of a patient in whom the PCNSL has relapsed in the cervical spinal cord and also in the liver.

## Background

Primary central nervous system lymphomas (PCNSLs) are rare diseases which account 1–2 % of non-Hodgkin lymphoma (NHL) and 3–5 % of primary brain tumor lesions (Ghesquières et al. 2009; Sierra Del Rio et al. 2009). Their diagnosis requires that any other location of systemic NHL is excluded (O’Neil et al. 1995). PCNSL is usually classified as a diffuse large B cell lymphoma. It occurs frequently as congenitally acquired or in immunocompromised patients. It commonly involves frontal lobes, corpus callum, or deep periventricular structures; may involve eyes, meninges or spinal cord. The treatment remains poorly codified and involves drugs crossing the blood–brain barrier which can be administered intrathecally or intravenously, associated or not with radiotherapy. Surgery with the increased risks of complications in deep locations is not needed outside of stereotactic biopsy for diagnostic purposes (Abrey et al. 2005). Relapses after treatment mostly occur in CNS, mainly leptomeningeal and ocular, <10 % are systemic. It report here a case of primary brain lymphoma relapse away from the primary lesion in the liver and in the CNS in an immunocompetent patient for whom the regular performance of positron emission tomography coupled to the scanner (PET/CT) and magnetic resonance imaging (MRI) has contributed to the diagnosis.

## Case report

**Fig. 1:**
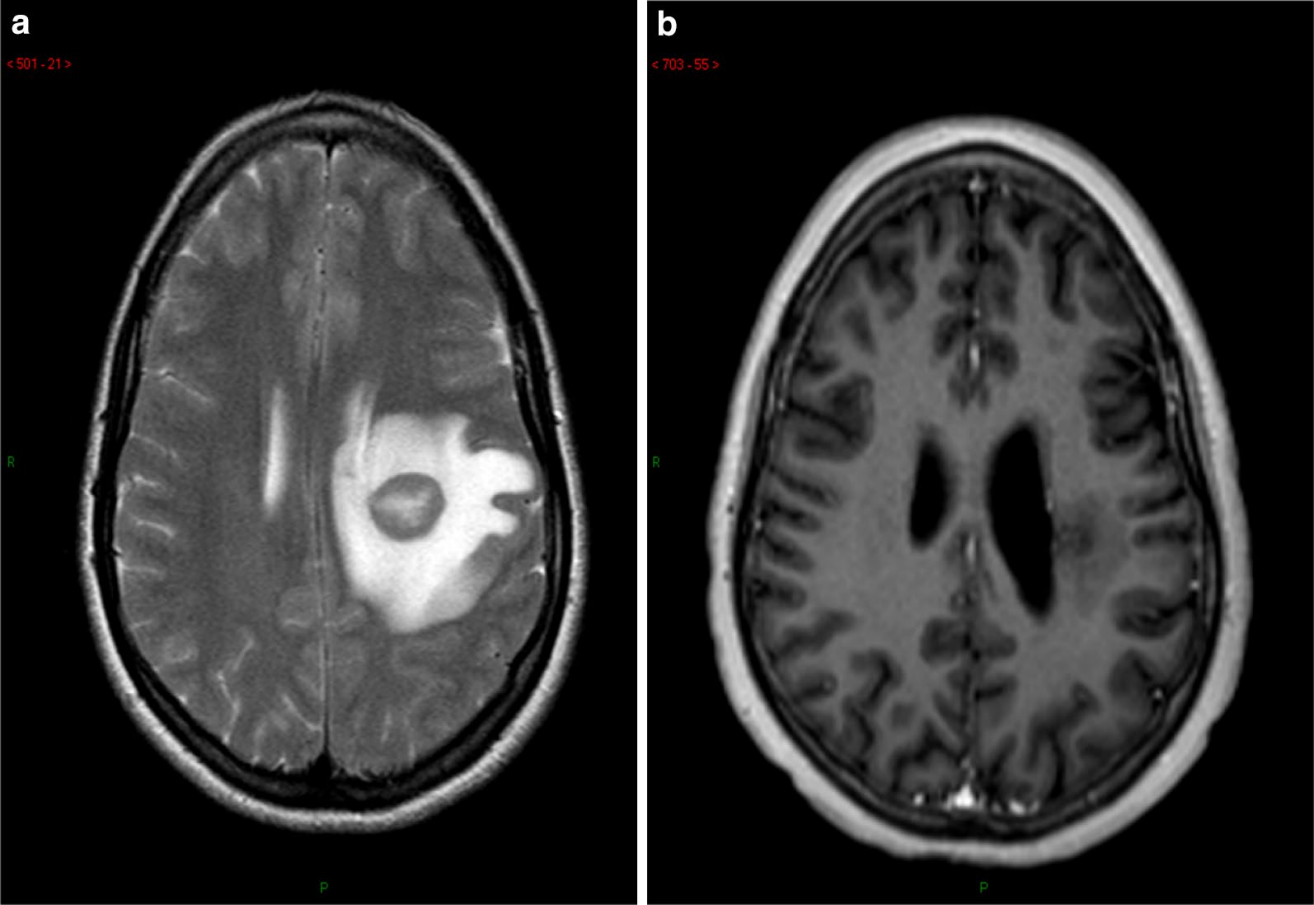
Brain MRI at diagnosis (**a**) and after two treatments by chemotherapy and radiotherapy (**b**)

A 20 years old patient with no past medical history came to the hospital for a feeling of weakness in the right upper which limb appeared a few days earlier. On admission general examination was normal, performance status was one with right hemiparesis. MRI reveals an oval left fronto-parietal lesion of 20 × 15 mm associated with significant peri-lesional edema (Fig. 1). A stereotactic biopsy was performed quickly, the histopathological analysis concluded an infiltration of the brain parenchyma by a diffuse large B-cells lymphoma, expressing the CD 20 antigen. The focus was then supplemented by serological testing which was all negatives (HIV, syphilitic, CMV, HBV, HCV), a bone marrow biopsy revealed no infiltration by lymphoma cell and was negative for CD20 or any other markers, immunophenotyping showed no monoclonal population and theslit-lamp eye examination was normal. PET/CT confirmed the absence of any lesion in favor of an extra-cerebral lymphomatous lesion.

Chemotherapy with high-dose methotrexate (3500 mg/m^2^) and cytarabine (2000 mg/m^2^) was administered.

**Fig. 2:**
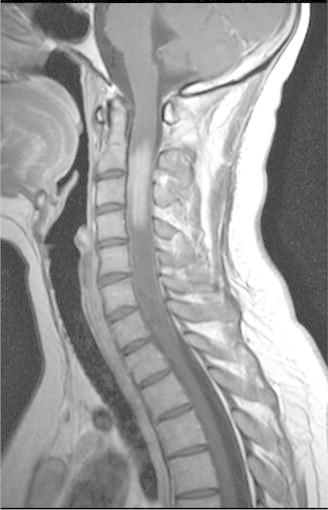
Medullary relapse of lymphoma

**Fig. 3:**
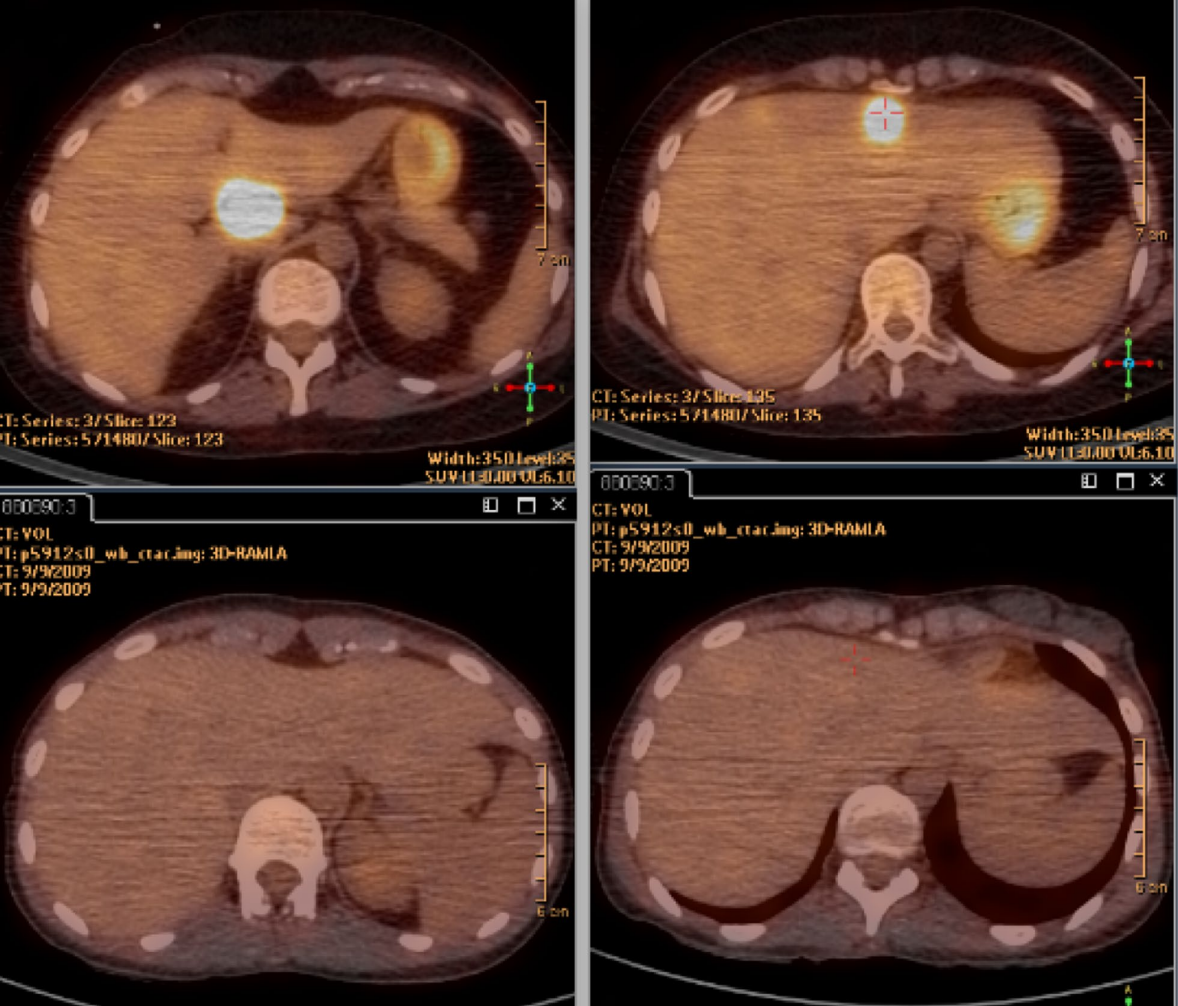
Comparaison of PET–CT at diagnosis (*low*) and liver relapse (*top*)

The evolution was firstly favorable but 2 months later neurologicals signs reappeared and a new MRI concluded an increase of the mass, 41 × 24 versus 32 × 18 mm at the last control). PET/CT has not found any systemic lesion. A second line of chemotherapy with ifosfamide (2000 mg/m^2^), coupled with radiotherapy was made with success but <1 month after the beginning of the treatment, the patient again presented with paresis of the right upper limb while the fronto-parietal brain injury declined. MRI of the whole spinal cord found an extending lesion from C2 to C4 (Fig. 2), which in this context, is suggestive of recurrence of lymphoma whereas no meningeal infiltration was found on lumbar puncture. It was decided to combine radiotherapy with temozolomide. PET/CT performed in conjunction confirmed the relapse, moreover, multiple liver lesions were found (Fig. 3). Liver biopsy found a recurrence of diffuse large B-cell lymphoma expressing the CD20 antigen with a high proliferation index (Ki67 in 95 % of tumor cells). Chemotherapy according to the protocol of the European Group of Lymphomas of the Adult (GELA): R-ACBVP (rituximab 375 mg/m^2^—adriamycin 75 mg/m^2^—cyclophosphamide 1200 mg/m^2^—vindesine 2 mg/m^2^—bleomycin 10 mg/m^2^—prednisone 60 mg/m^2^) was started but the patient died at the end of the second cycle of treatment.

## Discussion

The PCNSL of immunocompetent patient is an uncommon disease, it is estimated at 4 % of new diagnoses of CNS tumors. To diagnose CNSL, it is important to exclude other systemic NHL lesion, by conducting a number of tests as recommended by the GELA: a PET/CT or CT scans of the thorax, abdomen and pelvis, a bone marrow biopsy, a peripheral blood immunophenotyping and testicular ultrasound in men (O’Neil et al. 1995; Abrey et al. 2005). In patient, the diagnosis of PCNSL cannot be challenged both at the beginning and in the relapse, as all systemic exams were negative.

The prognosis of PCNSL is poor compared to other extranodal lymphomas, with a 5-year survival estimated between 20 and 40 %. Relapses occur between the first and second year in 30–60 % of patients in complete remission. In the literature survival after relapse varies from 2 to 4 months depending on the series (Jahnke et al. 2006).

PCNSL relapse occurs either in the original site but still confined to the CNS and exceptionally outside it. Jahnke et al. (2006) reported in a multicenter study 85 % of localized CNS relapse, 12 % systemic (nodal, musculoskeletal, testicular, kidney structure and para-kidney and liver) and the rest of both systemic and plants. If the brain parenchyma is the most common site of relapse of PCNSL, our patient’s first relapse occured within the CNS, but in an unusual site of CNS: in the cervical spine. Jahnke et al. reported 6 % of similar cases. In general, systemic relapse and in the liver in particular are very rare and few cases are reported.

Brain MRI, although not allowing a clear distinction between primary lesions and secondary brain lymphoma, is of paramount importance not only for diagnosis but also for monitoring the patient (Senocak et al. 2006). This is the strong clinical evidence that this exam result is important in diagnosis of the lymphomatous origin of the lesions observed in spite of the rarity of such evolution of the CNSL. Indeed, the cervical spinal cord although has a part of the CNS is not the usual location of PCNSL at diagnosis and his relapse.

In this case, the regular performance of PET/CT whole body has enabled us not only to confirm the nature of the primitive brain lymphoma (excluding when diagnosing any systemic involvement) but also it relapse in the cervical spine and liver. PET/CT has a resolution more efficient than conventional CT for the assessment of brain lymphoma as systemic lesions are identified much earlier (Mohil et al. 2008).

Although they have not been subject to confirmation by a phase III study, we opted for the first-line combination of high-dose MTX/cytarabine. In fact, these two agents have the advantage of crossing the blood–brain barrier in sufficient concentration to act on brain lymphoma lesions (Ferreri et al. 2002). Temozolomide for the choice of salvage treatment is related to the good results observed when using this drug in combination or not with radiotherapy for the treatment of relapsed or refractory PCNSL (Reni et al. 2007). The irradiation of the cervical spine, with or without chemotherapy, usually used in recurrent meningitis in our patient resulted in the disappearance of localized lesions between C2 and C4 (Rei and Ferreri 2001). Expression by lymphoma cells of CD 20 antigen drove us to use intravenous rituximab for the treatment of liver relapse according to the protocol R-ACBVP used in the treatment of high-grade NHL. A hope based on the use intrathecally this molecule in the treatment of relapsed CNSL, as suggested by the numerous cases published in recent years (Hong et al. 2009; Schulz et al. 2004; Watanabe et al. 2005; Pels et al. 2002).

## Conclusion

PCNSL, of which the usual locations are eye, meningeal and parenchymal, is rare and has a poor prognosis. It can also relapse at a distance from the primary lesion. Systemic relapse, poorly described so far, will receive a similar treatment to that of diffuse large cell NHL.
